# SYSTEMATIC REVIEW OF THE EFFECTS OF NON-VR EXERGAMING ON MOBILITY OUTCOMES OF OLDER ADULTS LIVING IN LONG-TERM CARE

**DOI:** 10.1093/geroni/igz038.1764

**Published:** 2019-11-08

**Authors:** Charlene H Chu, Renee Biss, Afroza Sultana, Amelie Gauthier-Beaupré, Arezoo Talebzadeh

**Affiliations:** 1 Lawrence S. Bloomberg Faculty of Nursing, University of Toronto, Toronto, Ontario, Canada; 2 York University, Toronto, Ontario, Canada; 3 McGill University, Montreal, Quebec, Canada; 4 University of Ottawa, Ottawa, Ontario, Canada; 5 OCAD, Toronto, Ontario, Canada

## Abstract

Introduction: Institutionalized older adults have high-rates of mobility decline resulting in reduced quality of life and increased dependency. Given the ageing population, there has been a proliferation of exergaming technology targeting older adults to maintain their physical activity (PA) levels and prevent decline. However, it is unclear if exergaming is effective to maintain or improve the PA of institutionalized older adults. Method: Four databases (MEDLINE/CINAHL/PsycINFO/Compendex) were systematically searched (key terms like “nursing homes”, exergaming”). Quantitative manuscripts examining the effects of exergaming on PA measures of institutionalized older adults published in English between 2006-present were eligible. Virtual reality was excluded from the search. No meta-analysis was conducted due to hetereogeneity of the results. Results: 11 studies were included from a search that yielded 208 results. The exergaming platforms that were used the most were the Kinect and Wii. The most commonly used PA measures were the Berg Balance Scale and the Timed-up-and-Go (n=4 studies) with no other measures being used in more than one study. Interventions ranged in exercise (e.g. cognitive-motor training, strength training, balance, etc), frequency, duration, and modality. Study designs were also heterogeneous. Articles were of very poor to poor quality. There was minimal reporting on adverse events. Older adults with cognitive impairment were commonly excluded. Challenges in current technology and studying this group are highlighted. Conclusion: Exergaming may be promising to maintain PA but more robust research is needed. More exergaming technology designed for long-term care to meet the specific complex needs of this population is warranted.

